# Molecular determinants of the adrenal gland functioning related to stress-sensitive hypertension in ISIAH rats

**DOI:** 10.1186/s12864-016-3354-2

**Published:** 2016-12-28

**Authors:** Larisa A. Fedoseeva, Leonid O. Klimov, Nikita I. Ershov, Yury V. Alexandrovich, Vadim M. Efimov, Arcady L. Markel, Olga E. Redina

**Affiliations:** 1 Institute of Cytology and Genetics, Siberian Branch of Russian Academy of Sciences, Novosibirsk, Russian Federation; 2 Novosibirsk State University, Novosibirsk, Russian Federation

**Keywords:** Stress-sensitive hypertension, Adrenal gland, Transcriptional profiling, RNA-Seq, PLS-DA, ISIAH rats

## Abstract

**Background:**

The adrenals are known as an important link in pathogenesis of arterial hypertensive disease. The study was directed to the adrenal transcriptome analysis in ISIAH rats with stress-sensitive arterial hypertension and predominant involvement in pathogenesis of the hypothalamic-pituitary-adrenal and sympathoadrenal systems.

**Results:**

The RNA-Seq approach was used to perform the comparative adrenal transcriptome profiling in hypertensive ISIAH and normotensive WAG rats. Multiple differentially expressed genes (DEGs) related to different biological processes and metabolic pathways were detected.

The discussion of the results helped to prioritize the several DEGs as the promising candidates for further studies of the genetic background underlying the stress-sensitive hypertension development in the ISIAH rats. Two of these were transcription factor genes (*Nr4a3* and *Ppard*), which may be related to the predominant activation of the sympathetic-adrenal medullary axis in ISIAH rats. The other genes are known as associated with hypertension and were defined in the current study as DEGs making the most significant contribution to the inter-strain differences. Four of them (*Avpr1a, Hsd11b2, Agt, Ephx2*) may provoke the hypertension development, and *Mpo* may contribute to insulin resistance and inflammation in the ISIAH rats.

**Conclusions:**

The study strongly highlighted the complex nature of the pathogenesis of stress-sensitive hypertension. The data obtained may be useful for identifying the common molecular determinants in different animal models of arterial hypertension, which may be potentially used as therapeutic targets for pharmacological intervention.

**Electronic supplementary material:**

The online version of this article (doi:10.1186/s12864-016-3354-2) contains supplementary material, which is available to authorized users.

## Background

The adrenal gland is known as a key organ playing an important role in the blood pressure regulation and hypertension development. The adrenal gland produces corticosteroid hormones and catecholamines that regulate a complex set of vital organismic functions including the stress control, water and sodium balance, cardiovascular system and the blood pressure (BP) level [1, 2]. That’s why the adrenal gland is an object of choice in a number of studies directed to elucidate the complex nature of hypertensive disease development or neuroendocrine profile related to stress response [3–5].

Different animal models of arterial hypertension help to analyze the transcriptome of the adrenal glands and to uncover common genetic mechanisms of hypertension across mammalian species that might, therefore, be pertinent to human hypertension too [3, 4, 6].

The ISIAH rat strain is a model of stress-sensitive arterial hypertension with predominant involvement of the neuroendocrine hypothalamic-pituitary-adrenocortical (HPA) and sympathetic adrenal systems (SAS) in pathogenesis of hypertension [7–9]. The peripheral plasma aldosterone concentration and secretion rates of corticosterone, 11-dehydrocorticosterone and deoxycorticosterone measured by the adrenal vein cannulation were significantly higher in the ISIAH rats as compared to control WAG rats [10]. The sympathetic adrenal medullary function assessed by measurement of the adrenal catecholamine content showed decreased concentrations of dopamine and norepinephrine, but significantly enhanced level of epinephrine in the adrenals of ISIAH rats [9]. It was suggested that the genetically determined enhanced stress responsiveness and hypertension development in ISIAH rats may be a result of the specificity of its adrenal gland function [10].

The differences in the transcription activity of several genes measured in the adrenal glands of hypertensive ISIAH and control WAG rats [9, 10] demonstrated that the selection of the ISIAH rat strain for the enhanced responsiveness to mild emotional stress could lead to accumulation of the genetic changes which may affect the hypertension development.

The goal of the current study was to compare the full transcriptome profiles of the adrenal glands from hypertensive ISIAH and normotensive WAG rats in order to identify the main pathways involved in the differences of their adrenal gland functions, and to define the differentially expressed genes (DEGs) which could make the largest contribution to the stress-sensitive hypertension development.

The current study of the comparative transcriptome profiling of the adrenal glands in hypertensive ISIAH and control WAG rats resulted in the detection of multiple DEGs related to different biological processes and metabolic pathways. The use of the partial-least squares discriminant analysis (PLS-DA) helped to reveal the top 10 DEGs associated with hypertension and making the most significant contribution to the inter-strain differences. Several of these DEGs may be considered as potential candidates for further studies directed to better understanding the mechanisms of hypertension development in the ISIAH rats.

## Results

Altogether, 12367 genes were defined as expressed in adrenal glands of ISIAH and WAG rats and were used in comparative expression analysis, which revealed 1113 DEGs. The complete listing of DEGs is given in Additional file 1. The hierarchical clustering based on Euclidean distance is shown in Additional file 2. More than half of the DEGs (619 genes, i.e., 55.6%) were upregulated in ISIAH rats.

The expression of 19 genes was detected in adrenal gland of only one rat strain (Additional file 3). Three of these genes (*Crp,* C-reactive protein, pentraxin-related; *Fabp1,* fatty acid binding protein 1, liver; *Ucp1,* uncoupling protein 1 (mitochondrial, proton carrier)) are known as related to hypertension development. Their expression was detected in adrenal gland of ISIAH rats but not in the WAG.

Altogether, the study revealed 76 DEGs annotated in Rat Genome Database (RGD) as related to hypertension (Table 1). Most of these genes (71.1%) were upregulated in hypertensive adrenal glands. Twenty three genes of those listed in Table 1 are known as associated with insulin resistance. Almost all of them (20 out of 23 genes) were upregulated in adrenals from ISIAH rats.

**Table 1:** Genes differentially expressed in ISIAH and WAG adrenal glands and referred to in Rat Genome Database as associated with hypertension

Gene symbol	Gene_ID	log2 (fold_change) ISIAH/WAG	Gene definition
*Ada*	24165	0.75	adenosine deaminase
*Adipoq* ^a^	246253	1.10	adiponectin, C1Q and collagen domain containing
*Adrb3* ^a^	25645	2.44	adrenergic, beta-3-, receptor
*Agt* ^a^	24179	2.28	angiotensinogen (serpin peptidase inhibitor, clade A, member 8)
*Alas1*	65155	0.75	aminolevulinate, delta-, synthase 1
*Alox5*	25290	−0.83	arachidonate 5-lipoxygenase
*Anxa3*	25291	0.69	annexin A3
*Aqp1*	25240	−0.49	aquaporin 1
*Atp1a2* ^a^	24212	1.26	ATPase, Na+/K+ transporting, alpha 2 polypeptide
*Avpr1a*	25107	1.12	arginine vasopressin receptor 1A
*Bche* ^a^	65036	1.32	butyrylcholinesterase
*C1qb*	29687	0.61	complement component 1, q subcomponent, B chain
*C3* ^a^	24232	2.06	complement component 3
*Cd36* ^a^	29184	1.04	CD36 molecule (thrombospondin receptor)
*Cdkn2b*	25164	−0.85	cyclin-dependent kinase inhibitor 2B (p15, inhibits CDK4)
*Cdo1*	81718	−1.20	cysteine dioxygenase, type I
*Cfh*	155012	0.76	complement factor H
*Col1a1*	29393	0.80	collagen, type I, alpha 1
*Crp* ^a^	25419	detected only in ISIAH rats	C-reactive protein, pentraxin-related
*Cx3cr1*	171056	0.75	chemokine (C-X3-C motif) receptor 1
*Cxcl10*	245920	−1.90	chemokine (C-X-C motif) ligand 10
*Cyba* ^a^	79129	0.68	cytochrome b-245, alpha polypeptide
*Dusp1*	114856	−0.94	dual specificity phosphatase 1
*Ednrb*	50672	0.82	endothelin receptor type B
*Egr1*	24330	−0.92	early growth response 1
*Entpd2*	64467	0.84	ectonucleoside triphosphate diphosphohydrolase 2
*Ephx2* ^a^	65030	4.37	epoxide hydrolase 2, cytoplasmic
*F2r*	25439	0.53	coagulation factor II (thrombin receptor
*F5*	304929	0.62	coagulation factor V (proaccelerin, labile factor)
*Fabp1*	24360	detected only in ISIAH rats	fatty acid binding protein 1, liver
*Fas*	246097	−0.80	Fas (TNF receptor superfamily, member 6)
*Fbn1*	83727	0.69	fibrillin 1
*Fmo3*	84493	0.57	flavin containing monooxygenase 3
*Fn1*	25661	0.76	fibronectin 1
*Gabbr1*	81657	1.11	gamma-aminobutyric acid (GABA) B receptor 1
*Gstm2*	24424	−0.60	glutathione S-transferase mu 2
*Gstp1*	24426	−0.53	glutathione S-transferase pi 1
*Hdac4*	363287	0.73	histone deacetylase 4
*Hmgb1*	25459	−0.84	high mobility group box 1
*Hmgcr* ^a^	25675	0.63	3-hydroxy-3-methylglutaryl-CoA reductase
*Hmox1* ^a^	24451	−0.69	heme oxygenase (decycling) 1
*Hp* ^a^	24464	1.10	haptoglobin
*Hsd11b2*	25117	−1.47	hydroxysteroid 11-beta dehydrogenase 2
*Hyal1*	367166	0.49	hyaluronoglucosaminidase 1
*Igf1*	24482	−2.12	insulin-like growth factor 1
*Itgav* ^a^	296456	−0.58	integrin, alpha V
*Loxl1*	315714	0.50	lysyl oxidase-like 1
*Lpl* ^a^	24539	0.52	lipoprotein lipase
*Mpo*	303413	4.46	myeloperoxidase
*Nov*	81526	−0.88	nephroblastoma overexpressed gene
*Pik3r1* ^a^	25513	0.50	phosphoinositide-3-kinase, regulatory subunit 1 (alpha)
*Postn*	361945	1.30	periostin, osteoblast specific factor
*Pparg* ^a^	25664	1.01	peroxisome proliferator-activated receptor gamma
*Prkcb* ^a^	25023	0.90	protein kinase C, beta
*Retn* ^a^	246250	2.26	resistin
*Rgs5*	54294	−0.85	regulator of G-protein signaling 5
*RT1-Ba*	309621	0.49	RT1 class II, locus Ba
*RT1-Db1*	294270	0.91	RT1 class II, locus Db1
*S100b*	25742	−1.72	S100 calcium binding protein B
*Serpina1* ^a^	24648	0.77	serpin peptidase inhibitor, clade A (alpha-1 antiproteinase, antitrypsin, member 1
*Serpine1* ^a^	24617	−2.04	serpin peptidase inhibitor, clade E (nexin, plasminogen activator inhibitor type 1), member 1
*Serpine2*	29366	2.19	serpin peptidase inhibitor, clade E, member 2
*Slc4a4*	84484	0.96	solute carrier family 4, sodium bicarbonate cotransporter, member 4
*Slc8a1*	29715	0.58	solute carrier family 8 (sodium/calcium exchanger), member 1
*Slc9a3*	24784	4.24	solute carrier family 9 (sodium/hydrogen exchanger), member 3
*Sod2*	24787	0.75	superoxide dismutase 2, mitochondrial
*Spp1*	25353	−0.56	secreted phosphoprotein 1
*Tacr2*	25007	0.84	tachykinin receptor 2
*Tap1*	24811	0.84	transporter 1, ATP-binding cassette, sub-family B (MDR/TAP)
*Tek*	89804	0.49	TEK tyrosine kinase, endothelial
*Tnc*	116640	1.74	tenascin C
*Trpc6*	89823	0.85	transient receptor potential cation channel, subfamily C, member 6
*Ucp1* ^a^	24860	detected only in ISIAH rats	uncoupling protein 1 (mitochondrial, proton carrier)
*Vcam1* ^a^	25361	0.78	vascular cell adhesion molecule 1
*Vip*	117064	−1.57	vasoactive intestinal peptide
*Xdh*	497811	−0.88	xanthine dehydrogenase

^a^- genes associated with insulin resistance; ISIAH and WAG – rat strains used in the study

Many of DEGs (166 genes) found in the current study are known as related to the metabolic diseases including hypercholesterolemia, hyperglycemia, hyperlipidemia, different types of hyperlipoproteinemias, and insulin resistance (Table 2).

**Table 2:** Genes differentially expressed in ISIAH and WAG adrenal glands and referred to in Rat Genome Database as associated with metabolic diseases

Gene symbol	Gene_ID	log2 (fold_change) ISIAH/WAG	Gene definition
*Abca1* ^a^ ^b^ ^d e^	313210	1.12	ATP-binding cassette, subfamily A (ABC1), member 1
*Abcg2*	312382	1.09	ATP-binding cassette, subfamily G (WHITE), member 2
*Acacb* ^a^	116719	0.98	acetyl-CoA carboxylase beta
*Acad9*	294973	0.63	acyl-CoA dehydrogenase family, member 9
*Acadsb*	25618	−0.71	acyl-CoA dehydrogenase, short/branched chain
*Acot2*	192272	−0.55	acyl-CoA thioesterase 2)
*Acp5*	25732	−1.54	acid phosphatase 5, tartrate resistant
*Ada* ^c^	24165	0.75	adenosine deaminase
*Adipoq* ^a^ ^d^	246253	1.10	adiponectin, C1Q and collagen domain containing
*Adrb3* ^a^	25645	2.44	adrenergic, beta-3-, receptor
*Adssl1*	684425	0.91	adenylosuccinate synthase like 1
*Agt* ^a^	24179	2.28	angiotensinogen (serpin peptidase inhibitor, clade A, member 8)
*Ahsg* ^a^ ^d^	25373	detected only in ISIAH rats	alpha-2-HS-glycoprotein
*Aif1*	29427	0.95	allograft inflammatory factor 1
*Ak1*	24183	−0.72	adenylate kinase 1
*Alas1*	65155	0.75	aminolevulinate, delta-, synthase 1
*Alpl* ^a^ ^b d^	25586	−1.32	alkaline phosphatase, liver/bone/kidney
*Anxa5*	25673	−0.97	annexin A5
*Aox1*	54349	1.74	aldehyde oxidase 1
*Apoc1* ^d^	25292	−0.90	apolipoprotein C-I
*Aqp1*	25240	−0.49	aquaporin 1
*Arsb*	25227	−0.49	arylsulfatase B
*Aspa*	79251	1.01	aspartoacylase
*Atp1a2* ^a^	24212	1.26	ATPase, Na+/K+ transporting, alpha 2 polypeptide
*Bche* ^a^ ^d^	65036	1.32	butyrylcholinesterase
*C1qa*	298566	0.77	complement component 1, q subcomponent, A chain
*C3* ^a^	24232	2.06	complement component 3
*Cartpt*	29131	−3.09	CART prepropeptide
*Casq1*	686019	−1.87	calsequestrin 1 (fast-twitch, skeletal muscle)
*Casq2*	29209	2.32	calsequestrin 2 (cardiac muscle)
*Ccl11* ^c^	29397	1.41	chemokine (C-C motif) ligand 11
*Cd36* ^a^	29184	1.04	CD36 molecule (thrombospondin receptor)
*Cfb*	294257	0.65	complement factor B
*Cfh*	155012	0.76	complement factor H
*Chek2* ^a^	114212	−0.92	checkpoint kinase 2
*Cidec*	500292	1.65	cell death-inducing DFFA-like effector c
*Col1a1*	29393	0.80	collagen, type I, alpha 1
*Cpox*	304024	0.50	coproporphyrinogen oxidase
*Crp* ^a^ ^d^	25419	detected only in ISIAH rats	C-reactive protein, pentraxin-related
*Cx3cr1*	171056	0.75	chemokine (C-X3-C motif) receptor 1
*Cxcl10*	245920	−1.90	chemokine (C-X-C motif) ligand 10
*Cxcl12*	24772	0.91	chemokine (C-X-C motif) ligand 12
*Cyba* ^a^	79129	0.68	cytochrome b-245, alpha polypeptide
*Cyp2e1*	25086	1.91	cytochrome P450, family 2, subfamily e, polypeptide 1
*Dab2*	79128	0.79	disabled homolog 2 (Drosophila)
*Dcaf12l1*	313296	−1.59	DDB1 and CUL4 associated factor 12-like 1
*Dcn*	29139	0.54	decorin
*Dgat1* ^a^ ^d^	84497	0.63	diacylglycerol O-acyltransferase 1
*Dusp1* ^c^	114856	−0.94	dual specificity phosphatase 1
*Ednrb*	50672	0.82	endothelin receptor type B
*Ehhadh*	171142	2.01	enoyl-CoA, hydratase/3-hydroxyacyl CoA dehydrogenase
*Entpd5*	314312	−1.00	ectonucleoside triphosphate diphosphohydrolase 5
*Ephx2* ^a^ ^b d e^	65030	4.37	epoxide hydrolase 2, cytoplasmic
*Ercc4*	304719	−1.12	excision repair cross-complementing rodent repair deficiency, complementation group 4
*F13a1*	60327	1.00	coagulation factor XIII, A1 polypeptide
*Fabp4* ^a^	79451	1.61	fatty acid binding protein 4, adipocyte
*Fam111a*	499322	3.85	family with sequence similarity 111, member A
*Fam126a*	499975	−0.72	family with sequence similarity 126, member A
*Fas*	246097	−0.80	Fas (TNF receptor superfamily, member 6)
*Fbn1*	83727	0.69	fibrillin 1
*Fgb*	24366	detected only in ISIAH rats	fibrinogen beta chain
*Fgg*	24367	1.47	fibrinogen gamma chain
*Fmo3*	84493	0.57	flavin containing monooxygenase 3
*Fn1*	25661	0.76	fibronectin 1
*Foxo1* ^a^	84482	−0.86	forkhead box O1
*Fus*	317385	0.65	fused in sarcoma
*Gabbr1*	81657	1.11	gamma-aminobutyric acid (GABA) B receptor 1
*Galns*	292073	0.81	galactosamine (N-acetyl)-6-sulfate sulfatase
*Gas6*	58935	0.50	growth arrest specific 6
*Gatm*	81660	−1.26	glycine amidinotransferase (L-arginine:glycine amidinotransferase)
*Gcgr*	24953	1.02	glucagon receptor
*Gfpt2*	360518	1.51	glutamine-fructose-6-phosphate transaminase 2
*Gk*	79223	−0.63	glycerol kinase
*Glrx*	64045	0.66	glutaredoxin (thioltransferase)
*Gria1*	50592	−2.39	glutamate receptor, ionotropic, AMPA 1
*Gstp1*	24426	−0.53	glutathione S-transferase pi 1
*Hap1*	29430	−0.61	huntingtin-associated protein 1
*Hdac4*	363287	0.73	histone deacetylase 4
*Hmgb1*	25459	−0.84	high mobility group box 1
*Hmgcr* ^a^ ^b d^	25675	0.63	3-hydroxy-3-methylglutaryl-CoA reductase
*Hmox1* ^a^	24451	−0.69	heme oxygenase (decycling) 1
*Hp* ^a^ ^b^ ^c d^	24464	1.10	haptoglobin
*Hsd11b2*	25117	−1.47	hydroxysteroid 11-beta dehydrogenase 2
*Hspa1a*	24472	0.70	heat shock 70kD protein 1A
*Hyal1*	367166	0.49	hyaluronoglucosaminidase 1
*Ifih1*	499801	0.69	interferon induced with helicase C domain 1
*Igf1*	24482	−2.12	insulin-like growth factor 1
*Il1r1*	25663	0.86	interleukin 1 receptor, type I
*Iscu*	288740	−0.65	iron-sulfur cluster scaffold homolog (E, coli)
*Isg15*	298693	0.95	ISG15 ubiquitin-like modifier
*Itga2*	170921	−1.29	integrin, alpha 2
*Itgav* ^a^ ^c^	296456	−0.58	integrin, alpha V
*Jak2* ^c^	24514	−0.63	Janus kinase 2
*Jak3*	25326	−0.57	Janus kinase 3
*Jam3*	315509	0.58	junctional adhesion molecule 3
*Kcnma1*	83731	0.77	potassium large conductance calcium-activated channel, subfamily M, alpha member 1
*Lcat* ^d^	24530	0.97	lecithin cholesterol acyltransferase
*Ldlr* ^b d^ ^e^	300438	0.93	low density lipoprotein receptor
*LOC689064*	689064	2.02	beta-globin
*Loxl1*	315714	0.50	lysyl oxidase-like 1
*Lpl* ^a^ ^d^ ^e^	24539	0.52	lipoprotein lipase
*Lrrk2*	300160	1.12	leucine-rich repeat kinase 2
*Lyz2*	25211	0.80	lysozyme 2
*Mgp*	25333	0.48	matrix Gla protein
*Mpo*	303413	4.46	myeloperoxidase
*Mpz*	24564	1.69	myelin protein zero
*Mt2A*	689415	−1.05	metallothionein 2A
*Myeov2*	681389	−0.53	myeloma overexpressed 2
*Myo5b*	25132	−1.13	myosin Vb
*Ndufaf2*	361894	−0.85	NADH dehydrogenase (ubiquinone) 1 alpha subcomplex, assembly factor 2
*Nefh*	24587	1.44	neurofilament, heavy polypeptide
*Nefm*	24588	−0.79	neurofilament, medium polypeptide
*Nr1d1*	252917	−0.57	nuclear receptor subfamily 1, group D, member 1
*Nucb2*	59295	−0.64	nucleobindin 2
*Oxct1*	690163	−0.86	3-oxoacid CoA transferase 1
*P2ry2*	29597	−1.31	purinergic receptor P2Y, G-protein coupled, 2
*Pah*	24616	−0.86	phenylalanine hydroxylase
*Pck1* ^c^	362282	3.83	phosphoenolpyruvate carboxykinase 1 (soluble)
*Pfkfb1*	24638	1.58	6-phosphofructo-2-kinase/fructose-2,6-biphosphatase 1
*Phgdh*	58835	−1.93	phosphoglycerate dehydrogenase
*Pik3r1* ^a^	25513	0.50	phosphoinositide-3-kinase, regulatory subunit 1 (alpha)
*Pla2g7* ^a^	301265	−0.97	phospholipase A2, group VII (platelet-activating factor acetylhydrolase, plasma)
*Plau* ^d^	25619	−0.78	plasminogen activator, urokinase
*Plin1*	25629	2.04	perilipin 1
*Postn*	361945	1.30	periostin, osteoblast specific factor
*Ppard* ^a^	25682	0.56	peroxisome proliferator-activated receptor delta
*Pparg* ^a^ ^d^	25664	1.01	peroxisome proliferator-activated receptor gamma
*Ppt1*	29411	0.48	palmitoyl-protein thioesterase 1
*Prkcb* ^a^ ^c^	25023	0.90	protein kinase C, beta
*Psmb9*	24967	0.47	proteasome (prosome, macropain) subunit, beta type 9 (large multifunctional peptidase 2)
*Ptprn*	116660	−0.75	protein tyrosine phosphatase, receptor type, N
*Rbp4*	25703	1.83	retinol binding protein 4, plasma
*Retn* ^a^	246250	2.26	resistin
*RGD1562200*	363471	0.84	patatin-like phospholipase domain-containing protein 4-like
*RT1-Ba* ^c^	309621	0.49	RT1 class II, locus Ba
*RT1-Da*	294269	0.80	RT1 class II, locus Da
*RT1-Db1*	294270	0.91	RT1 class II, locus Db1
*S100b*	25742	−1.72	S100 calcium binding protein B
*Scd1*	246074	2.16	stearoyl-Coenzyme A desaturase 1
*Scn1b*	29686	−0.56	sodium channel, voltage-gated, type I, beta
*Serpina1* ^a^	24648	0.77	serpin peptidase inhibitor, clade A (alpha-1 antiproteinase, antitrypsin, member 1
*Serpine1* ^a^	24617	−2.04	serpin peptidase inhibitor, clade E (nexin, plasminogen activator inhibitor type 1), member 1
*Slc11a1*	316519	0.69	solute carrier family 11 (proton-coupled divalent metal ion transporters), member 1
*Slc16a12*	309525	0.91	solute carrier family 16, member 12 (monocarboxylic acid transporter 12)
*Slc4a4*	84484	0.96	solute carrier family 4, sodium bicarbonate cotransporter, member 4
*Slc7a7*	83509	−0.69	solute carrier family 7 (amino acid transporter light chain, y%2BL system), member 7
*Slc8a1*	29715	0.58	solute carrier family 8 (sodium/calcium exchanger), member 1
*Slc9a3*	24784	4.24	solute carrier family 9 (sodium/hydrogen exchanger), member 3
*Sod2*	24787	0.75	superoxide dismutase 2, mitochondrial
*Sorbs1* ^a^	686098	−0.85	sorbin and SH3 domain containing 1
*Spg11*	311372	−0.55	spastic paraplegia 11 (autosomal recessive)
*Spp1*	25353	−0.56	secreted phosphoprotein 1
*Stat5b*	25126	−0.50	signal transducer and activator of transcription 5B
*Tap1*	24811	0.84	transporter 1, ATP-binding cassette, sub-family B (MDR/TAP)
*Thbs1*	445442	0.64	thrombospondin 1
*Tnc*	116640	1.74	tenascin C
*Trpc6*	89823	0.85	transient receptor potential cation channel, subfamily C, member 6
*Ttr*	24856	3.93	transthyretin
*Uchl5*	360853	−0.57	ubiquitin carboxyl-terminal hydrolase L5
*Ucp1* ^a^	24860	detected only in ISIAH rats	uncoupling protein 1 (mitochondrial, proton carrier)
*Unc13a*	64829	−1.01	unc-13 homolog A (C, elegans)
*Vcam1* ^a^ ^d^	25361	0.78	vascular cell adhesion molecule 1
*Vip*	117064	−1.57	vasoactive intestinal peptide
*Vldlr* ^b d^	25696	−0.61	very low density lipoprotein receptor
*Wfs1*	83725	1.77	Wolfram syndrome 1 homolog (human)
*Xdh*	497811	−0.88	xanthine dehydrogenase

Genes associated with: ^a^-insulin resistance; ^b^ *-* hypercholesterolemia; ^c^ - hyperglycemia; ^d^ -hyperlipidemia; ^e^ - hyperlipoproteinemias; ISIAH and WAG – rat strains used in the study

Sixty one transcription factor genes were differentially expressed in ISIAH and WAG adrenal glands (Table 3). Three of them are currently known as associated with hypertension development and 8 genes are referred to in RGD as related to metabolic diseases.

**Table 3:** Transcription factor genes differentially expressed in ISIAH and WAG adrenal glands

Gene symbol	Gene_ID	log2 (fold_change) ISIAH/WAG	Gene definition
*Ajuba*	85265	1.22	ajuba LIM protein
*Apbb1*	29722	0.52	amyloid beta (A4) precursor protein-binding, family B, member 1 (Fe65)
*Arhgap5*	299012	−0.58	Rho GTPase activating protein 5
*Bcl6*	303836	−1.16	B-cell CLL/lymphoma 6
*Cbfb*	361391	−0.66	core-binding factor, beta subunit
*Ccnc*	114839	−0.59	cyclin C
*Ccnl2*	298686	−0.72	cyclin L2
*Cebpa*	24252	0.85	CCAAT/enhancer binding protein (C/EBP), alpha
*Cnot3*	308311	0.60	CCR4-NOT transcription complex, subunit 3
*Creb3l1*	362165	1.22	cAMP responsive element binding protein 3-like 1
*Csrp2*	29317	−0.60	cysteine and glycine-rich protein 2
*Dab2*	79128	0.79	disabled homolog 2 (Drosophila)
*Egr1* ^a^	24330	−0.92	early growth response 1
*Ets2*	304063	−0.83	v-ets erythroblastosis virus E26 oncogene homolog 2 (avian)
*Etv1*	362733	−0.52	ets variant 1
*Fev*	246271	−0.92	FEV (ETS oncogene family)
*Foxo1* ^b^	84482	−0.86	forkhead box O1
*Fus*	317385	0.65	fused in sarcoma
*Grhl1*	313993	1.06	grainyhead-like 1 (Drosophila)
*Hcls1*	288077	0.63	hematopoietic cell specific Lyn substrate 1
*Hdac4* ^a^	363287	0.73	histone deacetylase 4
*Hes1*	29577	−0.84	hairy and enhancer of split 1 (Drosophila)
*Hltf*	295568	−0.58	helicase-like transcription factor
*Ifi204*	304988	1.05	interferon activated gene 204
*Irf7*	293624	1.64	interferon regulatory factor 7
*Irf9*	305896	0.77	interferon regulatory factor 9
*Junb*	24517	−1.58	jun B proto-oncogene
*Klhl6*	287974	1.00	kelch-like family member 6
*Lcor*	365462	−0.59	ligand dependent nuclear receptor corepressor
*Ldb3*	498587	1.30	LIM domain binding 3
*Mbd1*	291439	−0.81	methyl-CpG binding domain protein 1
*Mcm7*	288532	0.80	minichromosome maintenance complex component 7
*Mlxipl*	171078	−0.93	MLX interacting protein-like
*Mphosph8*	290270	−0.86	M-phase phosphoprotein 8
*Nfkbil1*	361794	0.62	nuclear factor of kappa light polypeptide gene enhancer in B-cells inhibitor-like 1
*Nfx1*	313166	0.50	nuclear transcription factor, X-box binding 1
*Nkx3-1*	305999	2.11	NK3 homeobox 1
*Nr1d1*	252917	−0.57	nuclear receptor subfamily 1, group D, member 1
*Nr4a3*	58853	1.20	nuclear receptor subfamily 4, group A, member 3
*Nrip1*	304157	−0.70	nuclear receptor interacting protein 1
*Pcaf*	301164	−0.54	p300/CBP-associated factor
*Pdlim3*	114108	1.06	PDZ and LIM domain 3
*Pml*	315713	0.54	promyelocytic leukemia
*Ppard* ^b^	25682	0.56	peroxisome proliferator-activated receptor delta
*Pparg* ^a b^	25664	1.01	peroxisome proliferator-activated receptor gamma
*Preb*	58842	0.52	prolactin regulatory element binding
*Pric285*	296474	0.50	peroxisomal proliferator-activated receptor A interacting complex 285
*Prpf4b*	291078	−0.58	PRP4 pre-mRNA processing factor 4 homolog B (yeast)
*Rbm43*	311020	0.80	RNA-binding protein 43
*Rere*	116665	−0.57	arginine-glutamic acid dipeptide (RE) repeats
*Smurf2*	303614	0.95	SMAD specific E3 ubiquitin protein ligase 2
*Stat5b*	25126	−0.50	signal transducer and activator of transcription 5B
*Tcf3*	171046	0.67	transcription factor 3
*Tfdp2*	300947	−0.78	transcription factor Dp-2 (E2F dimerization partner 2)
*Tgfb1i1*	84574	1.11	transforming growth factor beta 1 induced transcript 1
*Twist2*	59327	1.00	twist homolog 2 (Drosophila)
*Vgll3*	498038	1.66	vestigial-like family member 3
*Zbtb16*	353227	1.26	zinc finger and BTB domain containing 16
*Zfp281*	305083	−0.54	zinc finger protein 281
*Zfp292*	50552	−0.53	zinc finger protein 292
*Zmynd12*	313552	−0.96	zinc finger, MYND-type containing 12

Genes associated with: ^a^- hypertension; ^b^ - insulin resistance; ISIAH and WAG – rat strains used in the study

**Fig. 1:**
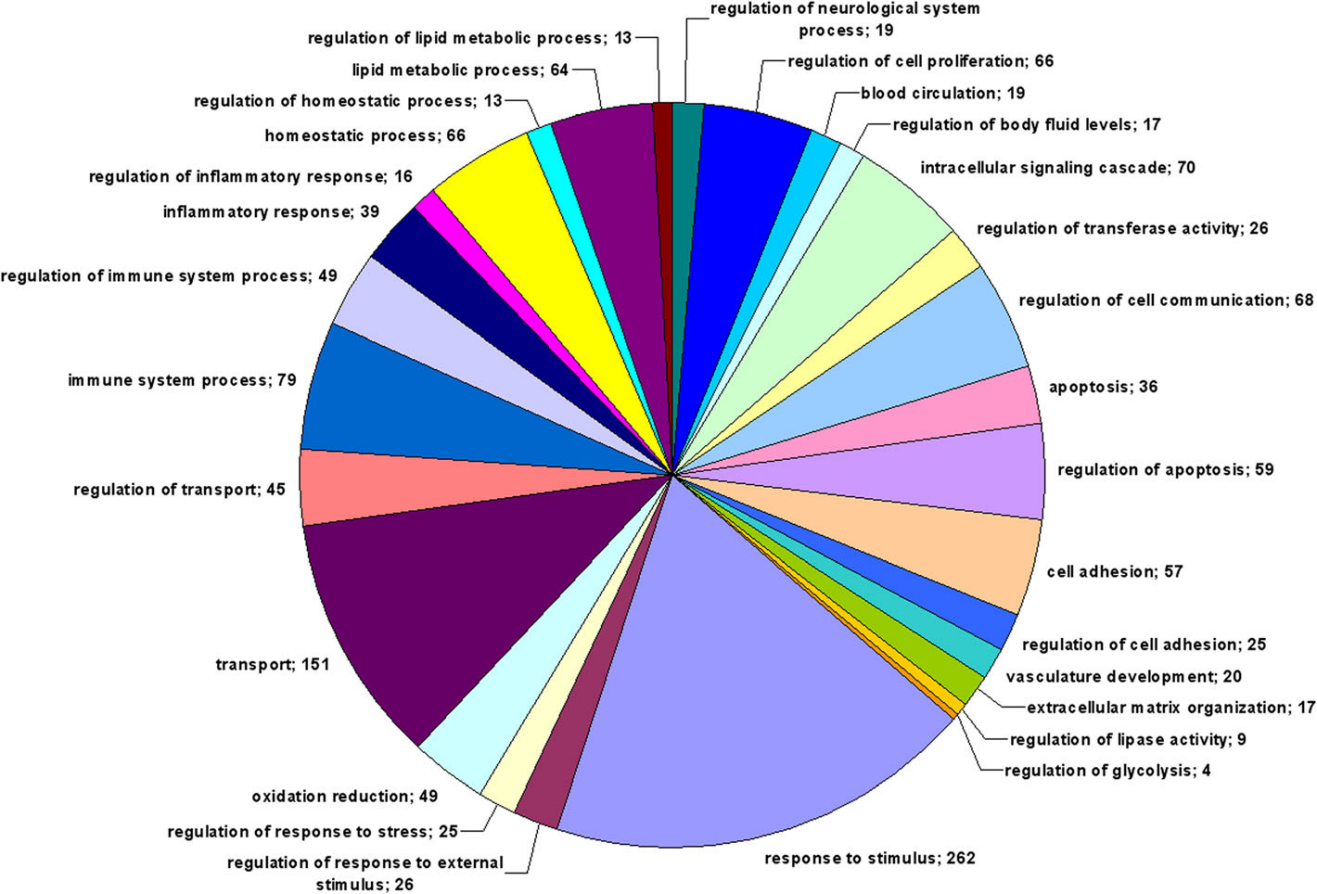
The main Gene Ontology (GO) terms for biological processes. Numerals represent the number of genes in the group

Gene Ontology (GO) terms for biological processes found to be significantly enriched are represented in Additional file 4. The groups of DEGs, which might be important for the development of the stress-sensitive hypertension, are given in bold in the file. The main groups are given in Fig. 1. The subgroups describing the specificity of the processes shown in Fig. 1 are represented in Additional file 5. The detailed information for genes in these groups is given in Additional file 6.

The most abundant group described by GO term ‘response to stimulus’ consisted of groups related to response to different stimuli - external stimulus, endogenous (hormone) stimulus, and stress, which were found to be among the most significantly enriched GO terms. The group of DEGs labelled ‘response to hormone stimulus’, consisted of subgroups of DEGs related to response to steroid hormone stimulus (and particularly to corticosteroid stimulus), response to growth hormone, and insulin stimuli. The response to stress was specified by the group of genes related to response to oxygen levels. Almost all genes (25 out of 27) in this group were related to response to hypoxia.

Several groups of DEGs related to BP control were found. These were: regulation of body fluid levels, blood circulation, blood coagulation, regulation of BP, regulation of angiogenesis and blood vessel size, regulation of smooth muscle cell proliferation and contraction.

Several other processes, which may play an important role in stress-sensitive hypertension development in ISIAH rats were: regulation of catecholamine secretion, glucose homeostasis, regulation of insulin-like growth factor receptor signaling pathway, oxidation reduction, calcium ion homeostasis, regulation of neurological system process (regulation of synaptic plasticity).

Multiple DEGs were related to transport (transport of lipids, cholesterol, and carboxylic acid) and regulation of transport. The differences of adrenal gland functioning in hypertensive ISIAH and normotensive WAG rats were also found to be under control of many genes involved in homeostatic process, lipid metabolic process, intracellular signaling cascade, cell adhesion and extracellular matrix organization, endocytosis, apoptosis, and the regulation of these biological processes.

The immune system process and its regulation were also among the most abundant and significantly enriched groups. Multiple DEGs were associated with inflammatory and adaptive, innate, and humoral immune responses.

Among the 15 significantly enriched (*p* < 0.05) KEGG (Kyoto Encyclopedia of Genes and Genomes) pathways identified in the current study, there were several associated with the function of immune system (Additional file 7). The other were related to complement and the blood coagulation cascades, PPAR signaling pathway, ECM-receptor interaction, focal adhesion, chemokine signaling pathway, glutathione metabolism. All of these pathways contained genes associated with hypertension and metabolic diseases.

**Fig. 2:**
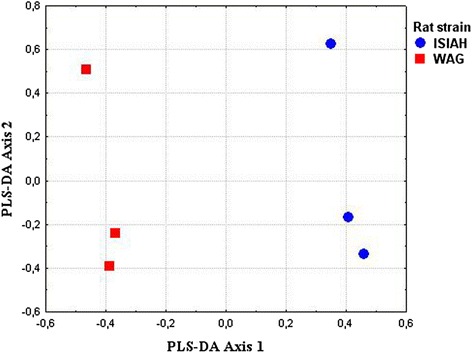
Axes maximizing the distances between ISIAH and WAG rats

**Fig. 3:**
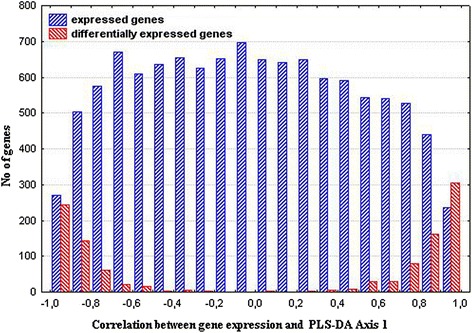
The correlation between genes expression and PLS-DA Axis 1. DEGs distribution is shown in *red*

**Fig. 4:**
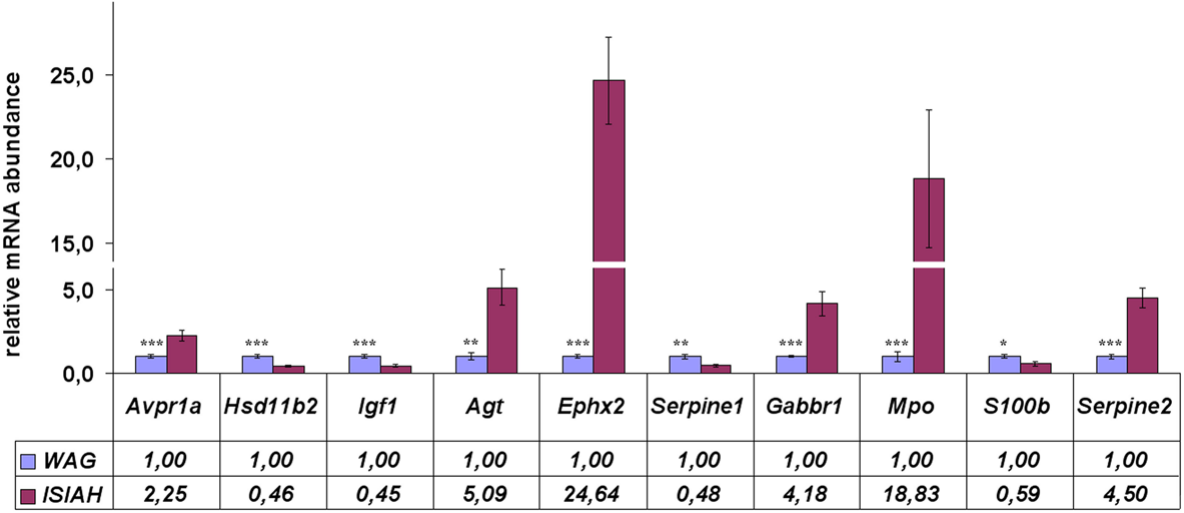
The relative mRNA abundance measured by qPCR. The significance of inter-strain difference is indicated by *p < 0.05, **p < 0.01, ***p < 0.001

The partial-least squares discriminant analysis (PLS-DA) was performed to identify the genes making the greatest impact to inter-strain differences. The constructed PLS-DA Axes maximized the distances between rats from two strains (Fig. 2), and the Pearson correlation calculated between gene expression and PLS-DA Axis 1 helped to determine the distribution of the genes along the axis representing the correlation between gene expression and PLS-DA Axis 1 (Fig. 3). The DEGs are shown in red in Fig. 3, and their polar position in the histogram assumes their contribution to the inter-strain differences. The 10 DEGs at the most polar position, which are known as associated with hypertension and showing greater than 2 fold differences in their level of transcription in the adrenal glands of ISIAH and WAG rats, were considered as the DEGs contributing the most to the inter-strain variations (Table 4). The differential transcription of these top 10 DEGs was validated by qPCR (Fig. 4). The comparison of the relative mRNA abundance between the RNA-Seq and qPCR measurements is represented in Additional file 8. The results obtained from the two methods were highly similar, with a calculated correlation coefficient of 0.99.

**Table 4:** The top 10 DEGs making the most significant contribution to the inter-strain differences and associated with hypertension

Gene symbol	Gene_ID	log2 (fold_change) ISIAH/WAG	Gene definition
*Agt*	24179	2.28	angiotensinogen (serpin peptidase inhibitor, clade A, member 8)
*Avpr1a*	25107	1.12	arginine vasopressin receptor 1A
*Ephx2*	65030	4.37	epoxide hydrolase 2, cytoplasmic
*Gabbr1*	81657	1.11	gamma-aminobutyric acid (GABA) B receptor 1
*Hsd11b2*	25117	−1.47	hydroxysteroid 11-beta dehydrogenase 2
*Igf1*	24482	−2.12	insulin-like growth factor 1
*Mpo*	303413	4.46	myeloperoxidase
*S100b*	25742	−1.72	S100 calcium binding protein B
*Serpine1*	24617	−2.04	serpin peptidase inhibitor, clade E (nexin, plasminogen activator inhibitor type 1), member 1
*Serpine2*	29366	2.19	serpin peptidase inhibitor, clade E, member 2

ISIAH and WAG – rat strains used in the study

## Discussion

The transcriptome profiling of the adrenal glands from ISIAH and WAG rats let to identify multiple DEGs and several pathways contributing to differences between the adrenal gland functions in ISIAH rats with stress-sensitive hypertension and normotensive controls.

The study revealed several genes with detected transcription in adrenal gland of only one rat strain. Three of them (*Crp, Fabp1,* and *Ucp1*), known as associated with hypertension, were expressed only in adrenal glands from hypertensive rats. However, the low levels of expression of these genes were reported in adrenals from normotensive Fischer 344 male rats, too [11]. So, the inter-strain differences in transcriptional activity of these genes shouldn’t be essential for hypertension development in ISIAH rats.

The specificity of the stress-sensitive hypertension may be seen from the functional annotation of DEGs performed in Database for Annotation, Visualization and Integrated Discovery (DAVID). The analysis showed that the group of DEGs described by GO term ‘response to stimulus’ was one of the most abundant. This could be a priori expected for the stress-induced models of hypertension, as the adrenal gland is a component of the HPA and sympathetic-adrenal medullary axes, which are both involved in neuroendocrine response to stress [12, 13]. However, in the current experiment, the rats were studied at rest condition. So, we may suggest that among the DEGs related to response to stimulus there should be those particular ones which define the predominant activation of the HPA and the sympathetic adrenal medullary axes in the pathogenesis of the hypertensive state in ISIAH rats selected for the enhanced BP in response to the mild emotional stress (0.5 h restriction in a small wire mesh cage) [7, 9].

Earlier in the study of the adrenal medulla transcriptome in Sprague-Dawley rats it was reported that multiple transcription factors were upregulated in response to the acute immobilization stress [5]. In the current experiment, the group of DEGs, associated with the GO term ‘response to stimulus’ in ISIAH rats, also contained multiple transcription factor genes most of which (13 out of 21) were upregulated. In the experiment with gene transcriptional profiling after acute immobilization stress in the adrenal medulla from the Sprague-Dawley rats [5] and in the current study we found 4 common transcription factor genes (*Egr1, Junb, Nr4a3,* and *Ppard*), with *Nr4a3* and *Ppard* being upregulated in both experiments. The orphan nuclear receptor, NOR-1 (also known as NR4A3) was reported as a target of beta-adrenergic signaling in skeletal muscles [14]. Taking all the information together, we may hypothesize that the enhanced transcriptional activity of *Nr4a3* may be related to the predominant activation of the sympathetic-adrenal medullary axis in ISIAH rats. To our knowledge, the role of the sympathetic nervous system in activation of *Ppard* has not been described up to date, however, its important role may be expected from the study of the acute immobilization stress response of the adrenal glands in Sprague-Dawley rats [5].

The adrenal medullary tissue contributes to maintain body homeostasis in stressful environment via the release of catecholamines into circulatory system in response to splanchnic nerve activation [15]. The acetylcholine released by the sympathetic splanchnic nerves activates neuronal-type nicotinic acetylcholine receptors (nAChRs) on the membrane of chromaffin cells which liberate catecholamines into the bloodstream in preparation for the fight and flight reactions [16]. In the current study several DEGs involved in the regulation of catecholamine secretion were found (Additional file 6), including *Chrna4* (cholinergic receptor, nicotinic, alpha 4). However, *Chrna4* was downregulated in ISIAH adrenals. Besides, one more gene in this group, *Cartpt* (CART prepropeptide) known as activating sympathoadrenal outflow [17], was also decreased. These findings suggest the involvement of the *Chrna4* and *Cartpt* genes in compensatory mechanism directed to attenuate the catecholamine release by the adrenals of ISIAH rats.

The predominantly increased effectiveness of the HPA axis in the ISIAH rats may be related to the DEGs participating in response to hormonal stimuli. Most of the DEGs in this group (16 out of 25) were upregulated in the adrenal glands from ISIAH rats (Additional file 6), and about half of them are known as associated with hypertension. Three of these DEGs (*Avpr1a*, arginine vasopressin receptor 1A; *Hsd11b2,* hydroxysteroid 11-beta dehydrogenase 2; and *Igf1*, insulin-like growth factor 1) were reckoned among the top 10 DEGs making the most significant contribution to the inter-strain differences (Table 4).

V1a receptor (*Avpr1a*) plays an important role in the basal arterial BP maintenance by regulation of circulating blood volume and baroreflex sensitivity [18]. Vasopressin is a potent autocrine/paracrine regulator of mammal adrenal functions. V1a receptor is expressed both in adrenal cortex and adrenal medulla. In the adrenal cortex V1a receptor triggers both steroid secretion and cortical growth [19]. Besides, V1a receptor is present in vascular smooth muscles and is responsible for the classical vasopressor action of vasopressin [20]. The *Avpr1a* upregulation in the ISIAH adrenal glands may indicate the exaggerated effects on multiple adrenal functions in ISIAH rats.

Our previous studies confirmed the reduced activity of 11β-hydroxysteroid dehydrogenase type 2 (11β-HSD2) in adrenal glands of ISIAH rats [21] and in peripheral blood plasma [10]. It was shown that the loss-of-function mutations or inhibition of 11β-HSD2 results in overstimulation of the mineralocorticoid receptor by glucocorticoids and causes salt-sensitive hypertension [22]. Taking into account the decreased level of *Hsd11b2* transcription and its protein activity in adrenal glands and other tissues of ISIAH rats, which resulted in decrease of 11-dehydrocorticosterone/corticosterone ratio in peripheral blood plasma, we may suggest the importance of this mechanism in stress-sensitive hypertension development, too.

The decreased transcription of *Igf1* in ISIAH adrenals is in a good agreement with the observation that IGF1 expression may be significantly decreased in the presence of hypertension [23]. An increase in the IGF1 production was reported in rats undergoing the compensatory growth of the adrenal gland following the unilateral adrenalectomy [24]. As the weight of the adrenal glands is significantly higher in ISIAH rats as compared to WAG rats [25], we suggest that the decreased transcription of *Igf1* in ISIAH adrenals may be adaptive.

The data of the current study revealed also many other DEGs associated with hypertension and metabolic diseases, the main feature of which is insulin resistance. Adrenocortical dysregulation is considered as a major player in insulin resistance and onset of obesity [26]. It is believed that insulin resistance is directly correlated with the severity of hypertension [27] and may account for the etiology of essential hypertension in as many as half of the patients with the disease [28]. In the current study, three DEGs associated with both hypertension and insulin resistance (*Agt,* angiotensinogen; *Ephx2*, epoxide hydrolase 2; and *Serpine1,* serpin peptidase inhibitor, clade E (nexin, plasminogen activator inhibitor type 1), member 1) were put on the list of the top 10 DEGs making the most significant contribution to the inter-strain differences (Table 4).

Angiotensinogen is the substrate of renin and the precursor of the angiotensin peptides having the powerful vasoconstrictive properties. The activation of renin-angiotensin system (RAS) is considered not only as a main hypertensive system, but also as a key factor triggering reactive oxygen species production, oxidative stress, endothelial dysfunction and hypertension development [29]. The elevated transcription of the *Agt* gene in ISIAH adrenal glands points out the involvement of the adrenal tissue RAS in the development of stress-sensitive hypertension. However, our data differ from those previously reported for spontaneously hypertensive rats (SHR). It was shown that adrenal angiotensinogen mRNAs were lower in SHR than in control WKY rats at 14 weeks of age [30]. This discrepancy is probably one of the features distinguishing the mechanism of hypertension development in ISIAH rats from that in SHRs.

*Ephx2* encodes the soluble epoxide hydrolase (sEH) that metabolizes the epoxyeicosatrienoic acids, which produce vasorelaxation and exert anti-inflammatory and pro-fibrinolytic effects [31]. sEH was linked to hypertension in the studies on different animal models of the disease: spontaneous [32], angiotensin II-induced [33], and programmed hypertension [34]. Soluble epoxide hydrolase deficiency improves glucose homeostasis in a model of insulin resistance [35]. Taking into account the above information, we may suggest that *Ephx2* activation may contribute to disease development in ISIAH rats.

The plasminogen activator inhibitor 1 (PAI1 or SERPINE1), known as coagulation marker, has been found to correlate with all components of the insulin resistance syndrome, and can be considered as a true component of the metabolic syndrome [36]. Increased plasma PAI1 may be involved in the occurrence of micro-vascular complications and increased risk of atherosclerosis [37]. The inhibition of PAI1 results in reduction of cell adhesion and cellular proliferation, particularly in reduction of angiogenesis [38]. So, decreased transcription of the *Serpine1* in ISIAH adrenals may work against the excessive development of angiogenesis and micro-vascular complications in stress-sensitive hypertension.

As long as the PLS regression method is commonly used for biomarker selection in metabolomic [39] and gene expression [40] studies, the other DEGs listed in the Table 4 (*Gabbr1,* gamma-aminobutyric acid B receptor 1; *Mpo,* myeloperoxidase; *S100b*, S100 calcium binding protein B; and *Serpine2,* serpin peptidase inhibitor, clade E, member 2) may also be indicated as deserving a high priority in future investigations of molecular mechanisms of the stress-sensitive hypertension. Their possible contribution to the disease development in ISIAH rats is discussed below.

Metabotropic GABAB receptors (GABABRs) abundantly expressed at inhibitory and excitatory synapses are mostly studied in the brain, where they play an important role in modulating synaptic transmission by their presynaptic inhibitory effects on calcium channels and postsynaptic activating effects on potassium channels [41, 42]. It was also shown that activation of GABABRs protects neurons from apoptosis via IGF1 receptor transactivation [43]. However, gamma-aminobutyric acid (GABA) is produced not only in the brain, but also in endocrine cells including rat adrenal medullary chromaffin cells. Since there are no GABAergic nerve fibers in the adrenal medulla, GABA may function as a para/autocrine factor [44]. The functional role of the elevated transcription of *Gabbr1* in adrenal glands is not known and has to be studied, as it might be essential for stress-sensitive hypertension development in ISIAH rats.

Myeloperoxidase (MPO) delays neutrophil apoptosis and prolongs inflammation [45]. The activation of MPO may contribute to the development of obesity and obesity-associated insulin resistance [46]. So, it may be expected that the elevated *Mpo* transcription found in ISIAH adrenals in the current study may contribute to insulin resistance and inflammation in ISIAH rats, too.

*S100b* expression is studied mostly in the central nervous system, where the role of S100 beta is related to the development and maintenance of neuronal function [47]. S100 beta may influence the cell survival in a concentration-dependent manner [48]. Recently, it was reported that the decreased expression of S100 beta may be associated with the neuroprotective mechanism against acute stress [49]. So, the role of the *S100b* decreased transcription in adrenal glands of ISIAH rats may be associated with the sympathetic nervous system regulation of the stress-sensitivity in ISIAH rats.

*Serpine2* encodes serine (or cysteine) proteinase inhibitor, clade E, member 2 (or protease nexin1, PN-1) which is associated with the negative regulation of blood coagulation [50, 51].

As it is seen from the above discussion, the top 10 DEGs making the most significant contribution to the inter-strain differences (Table 4) possess different functional properties and may contribute to many physiological mechanisms possibly related to hypertension development in ISIAH rats. Four of these DEGs (*Agt, Avpr1a, Ephx2,* and *Hsd11b2*) were related to GO term group 'regulation of BP'. Among the other members of this group there was the transcription factor *Pparg*.

The pathway enrichment analysis in KEGG database showed that the PPAR (peroxisome proliferator-activated receptor) signaling pathway was among the most significantly enriched in the current study. The DEGs related to this pathway were mostly upregulated in ISIAH adrenal glands (Additional file 7). Among the upregulated DEGs there were 6 genes associated with hypertension, including the *Adipoq* and *Lpl* genes encoding adiponectin and lipoprotein lipase, which are recognized as an indicators for PPAR-gamma activation [52–54]. In the current study two genes (*Ppard* and *Pparg*) encoding the members of the PPAR subfamily of nuclear receptors were upregulated.

The physiological role of PPARs is related to lipid metabolism and energy homeostasis [53]. PPAR-gamma has been implicated in the pathology of numerous diseases including insulin resistance, diabetes, atherosclerosis and hypertension [55, 56]. PPAR-gamma activation attenuates insulin resistance and inflammation [57, 58]. PPAR-delta activation ameliorates obesity and insulin resistance [59], and has been considered as a potential therapeutic target in treatment of lipid-related disorders, including dyslipidemia and diabetes [60, 61].

Earlier it was shown that the hypertension development in ISIAH rats is accompanied by dislipidemia, increased glucose content, increased body weight, and enhanced DNA-binding activity of several transcription factors including PPARs in liver. These data suggested the development of metabolic syndrome in ISIAH rats [62]. Probably, the elevated transcription of *Pparg* and *Ppard* in the adrenal gland of ISIAH rats plays adaptive role and is directed to the attenuation of the processes leading to the metabolic syndrome development.

Both GO and KEGG analyses indicated the high impact of the immune system processes on the formation of the interstrain differences in ISIAH and WAG rats (Fig. 1 and Additional file 7). Multiple DEGs associated with GO term ‘immune system process’ are annotated in RGD as associated with hypertension. The important role of inflammation and immunity in development of the stress-sensitive hypertension was already highlighted in our previous comparative studies of genome-wide transcriptome analyses of hypothalamus and renal cortex from ISIAH and WAG rats [63, 64]. A growing body of research supporting a role of inflammation and immunity in hypertension was recently summarized in multiple reviews [65–72]. Many of the authors reviewing the problem consider that cells of both the innate and adaptive immune system contribute to end-organ damage and dysfunction in hypertension, and the molecular determinants of the immune cells activation may be a putative therapeutic targets to reduce end-organ damage and prevent pathological consequences of hypertension [68, 69, 73]. The results of our study are in a good agreement with these opinions and may be useful to define the common molecular determinants, which may be recognized as potential targets for therapy and prevention of hypertensive disease.

## Conclusion

Recently, the molecular studies of the pathogenesis of genetic hypertension strongly highlighted the complex nature of the disease. The current study of the comparative transcriptional profiling of the adrenal glands in ISIAH rats with the stress-sensitive arterial hypertension and control WAG rats resulted in detection of multiple DEGs related to different endocrine, inflammatory, neural, and metabolic processes and pathways. The discussion of the results helped to prioritize the following genes.

Two transcription factor genes (*Nr4a3* and *Ppard*) were found to be common and upregulated both in adrenal of ISIAH rats and in the adrenal medulla from the Sprague-Dawley rats after acute immobilization stress. We suggest that the upregulation of these genes may be related to the predominant activation of the sympathetic-adrenal medullary axis in ISIAH rats; however, their real contribution to the hypertensive phenotype remains to be demonstrated.

The use of the PLS-DA helped to reveal a number of DEGs making the most significant contribution to the inter-strain differences. The discussion of ten of them known as associated with hypertension demonstrated that four of these genes (*Avpr1a, Hsd11b2, Agt, Ephx2*) may provoke the hypertension development, and *Mpo* may contribute to insulin resistance and inflammation in ISIAH rats. These DEGs may be considered as the most promising candidates for further studies of the mechanisms underlying the stress-sensitive hypertension development.

It was not possible to discuss the functional roles for all the DEGs found in the current study. The differential expression of the genes not necessary must be related to hypertensive phenotype. So, the attention was mostly paid to the discussion of the DEGs already known as associated with hypertension, which could be considered as the most potentially interesting candidates for further studies of the mechanisms underlying the stress-sensitive hypertension development. However, the list of genes associated with hypertension is permanently expanding. Thus, we can’t exclude that the other genes found to be differentially expressed in ISIAH and WAG adrenal glands may also influence the development of hypertensive phenotype.

The results of the current study may be useful to identify the common molecular determinants in different animal models of arterial hypertension and to define the potential targets for therapy and prevention of hypertensive disease.

## Methods

### Animals

The study was performed using the hypertensive ISIAH/Icgn and normotensive WAG/GSto-Icgn rat strains. The rats from both strains were bred in the Center for Genetic Resources of Laboratory Animals at the Institute of Cytology and Genetics, SB RAS, Novosibirsk, Russia (Identification numbers in the list of National Animal Facilities of Russia RFMEFI61914X0005 and RFMEFI62114X0010).

The ISIAH rat strain (Inherited Stress-Induced Arterial Hypertension) is a rat model with the genetically determined exaggerated sensitivity to stressful stimuli [9]. The ISIAH rats were selected for a strong elevation of the systolic arterial blood pressure (SABP) in response to a brief emotional stress. To cause the emotional stress the animal was kept for 30 min in a small cylindrical wire mesh cage [7, 8]. This procedure leads to 20–25 mmHg elevation of SABP in ISIAH rats and doesn’t cause the significant changes of SABP in WAG rats used as a normotensive control. Both ISIAH and WAG rat strains derived from outbred Wistar rats. The process of ISIAH rat strain selection for the dramatic increase of SABP during mild emotional stress was accompanied by the elevation of the SABP at rest condition, which is about 175.0 ± 3.5 mmHg in males and 165.0 ± 3.0 mmHg in females from the current population. The high degree of genetic homogeneity of the ISIAH strain was confirmed by the DNA fingerprinting approach [74].

All rats were kept under the standard environmental conditions with ad libitum access to food and water. Animals were individually caged a week before the SABP measurement, which was done indirectly by the tail-cuff method with the use of short-term ether anesthesia. The preliminary work showed that the blood pressure measured in the ether anesthetized rats is close to the measures made in the unanesthetized rats after many days of adaptation to the procedure of indirect tail-cuff method as well as to the blood pressure levels measured in the home-cage drectly trough indwelling arterial catheter.

The RNA-Seq experiments were conducted on ISIAH and WAG males aged 3-month old. Each experimental group consisted of three rats. Their SABP was 171.7 ± 1.2 mmHg and 116.3 ± 1.9 mmHg in ISIAH and WAG males, correspondingly. Six days after SABP measurement, rats were decapitated, and their left adrenal glands were immediately removed and stored in RNA Later (Qiagen, Chatsworth, CA) at −70 °C. The relative amount of target mRNA was measured by semi-quantitative real-time PCR (qPCR) in the left adrenal glands from 3-month old ISIAH and WAG male rats. Each group consisted of seven rats. Their SABP was measured as described above. It was 174.3 ± 1.3 mmHg in ISIAH and 122.1 ± 1.8 mmHg in WAG rats. The rats were also decapitated 6 days after measurement of SABP, and their left adrenal glands were rapidly removed, frozen and stored at −70 °C until use.

The animal experiments protocols received approval of the Institute’s Animal Care and Use Committee.

### RNA-Seq analysis

The technological part of the RNA-seq analysis was performed in JSC Genoanalytica (Moscow, Russia). The mRNA from the samples of agrenal glands was extracted using Dynabeads mRNA Purification Kit (Ambion, USA). NEBNext mRNA Library Prep Reagent Set for Illumina (NEB, USA) was used to construct the cDNA libraries following the manufacturer’s protocol. The single-end sequencing of the cDNA libraries was carried out on Illumina HiSeq1500 Sequencing System (Illumina Sequencing, San Diego, USA) with read length of 50 bases. All samples were run as biological replicates. The sequencing data after adapter trimming and low-quality sequence removal were mapped to the RGSC Rnor_5.0\rn5 reference genome with the use of Tophat2 aligner [75]. CollectRnaSeqMetrics from the Picard tools suit (http://broadinstitute.github.io/picard/) was used to collect the quality metrics of the mapped data (Additional file 9). The Cufflinks program was employed to count gene expression levels in FPKM (fragments per kilobase of transcript per million mapped reads). Gene annotation was based on NCBI Gene/RefSeq database. A gene was defined as being expressed if it has successfully passed the Cufflinks statistical testing and was assigned to test status ‘OK’. Cuffdiff was used to identify the genes with differential expression under a false discovery rate (FDR) threshold of 0.05 [76]. The RNA-Seq data were deposited in the NCBI SRA database under the Accession number: PRJNA299102.

### Functional annotation

The DAVID (The Database for Annotation, Visualization and Integrated Discovery) tool (http://david.abcc.ncifcrf.gov/) was employed for functional annotation of DEGs [77, 78]. The *Rattus norvegicus* genome was utilized as the background list for the over-representation analysis. The Gene Ontology option was used to identify the significantly (*p* < 0.05) enriched biological processes. The Kyoto Encyclopedia of Genes and Genomes (KEGG, http://www.genome.jp/kegg/) Pathway Database was used to identify the significantly (*p* < 0.05) enriched metabolic pathways. The annotation of DEGs in Rat Genome Database (RGD, http://rgd.mcw.edu/) helped to reveal the genes associated with hypertension and metabolic diseases. The DEGs were annotated in GenBank (http://www.ncbi.nlm.nih.gov/gene/), an atlas of combinatorial transcriptional regulation in mouse and man [79], and Panther classification system (http://www.pantherdb.org/) [80] to reveal those encoding the transcription factors.

### qPCR

The extraction of the total RNA was carried out with the use of the TRI reagent RNA isolation protocol (Molecular research center, USA), and the residual genomic DNA was removed from the total RNA samples by DNase I (Promega, USA) treatment, following the manufacturer’s instructions.

The reaction mixture for reverse transcription contained reverse transcription buffer (Vektor-Best, RF), 0.25 nmol of random nonanucleotide primers (Biosan, RF), 0.4 mM dNTPs, 3 μg of RNA, and 40 units of MoMLV (Vektor-Best, RF) in the total volume of 50 μl. The protocol for cDNA synthesis was as follows: 1 h at 37 °C, 30 min at 42 °C, and 10 min at 50 °C. The enzyme was inactivated by heating the reaction mixture during 5 min at 75 °C.

qPCR was carried out in a final volume of 20 μl containing a master mix with SYBR Green, 0.15 mM of each forward and reverse primers, the cDNA template, and 1 unit of HotStart Taq polymerase (Vektor-Best, RF). The *Ppia (*peptidylprolyl isomerase A) was used as a reference gene. Primer sequences and their characteristics are represented in Additional file 10.

The iCycler iQ4 Real-Time PCR Detection System (Bio-Rad Laboratories, USA) was used to run qPCR. The reaction was started at 94 °C for 1 min and followed by 40 cycles of 15 s at 94 °C, 20 s at primer’s annealing temperatures listed in Additional file 10, 20 s at 72 °C, and fluorescence signal acquisition (10 s). The melting curve was generated in the range of 65 °C to 94 °C. Relative transcript levels were determined with the use of standard-curve quantitation method [81]. The aliquots from each of the synthesized cDNA samples were pooled and used as the standard cDNA. The qPCR was run using the same cDNA samples with primers for the target gene and for the reference gene, which were loaded onto the plate as four replicates per cDNA sample, and the standard cDNA dilutions (1 : 1, 1 : 4, 1 : 16, and 1 : 64) with the primers for the target gene (two replicates), and for the reference gene (two replicates) loaded onto the same plate. iCycler iQ4 Real-Time PCR Detection System software was used to build the calibration curves for calculation of the relative amount of cDNAs.

The value for the target gene was normalized against the value obtained for the reference gene. The relative mRNA abundance was calculated as a ratio of the normalized mRNA level calculated for the experimental ISIAH samples to the normalized mRNA level obtained for the samples from control WAG rats, which was set a value of 1.

### Statistical methods

Statistical significance for qPCR data was calculated by Student’s *t*-test. A *p* value < 0.05 was considered significant. Data were expressed as means and standard errors of means (M ± SEM).

The data (FPKM values) obtained from RNA-Seq were log transformed, centered, and normalized. The principal coordinates analysis based on Euclidean metric distances was employed to scale the data sets, and PLS-DA was used to construct the PLS-DA Axes maximizing the distances between ISIAH and WAG rats. Then, the Pearson correlation was explored to determine a set of variables (i.e. expressed genes) that maximize the covariance between gene expression in ISIAH and WAG rats and fixed dummy matrix representing group membership [82] for rats from different strains. These procedures helped to define the genes showing the most deviation along the axis representing the correlation between gene expression and PLS-DA Axis 1, which were assumed as genes contributing the most to inter-strain variations. The Pearson correlation between mean values (represented as log2(fold_change)ISIAH/WAG) obtained by RNA-Seq and qPCR for 10 genes (*Agt, Avpr1a, Ephx2, Gabbr1, Hsd11b2, Igf1, Mpo, S100b, Serpine1, Serpine2*) was used to count the correlation coefficient between the results derived from these methods.

## Additional files

Additional file 1: Genes differentially expressed in adrenal glands of 3 month old hypertensive ISIAH and normotensive control WAG rats. (XLS 214 kb)
Additional file 2: Heatmap of the differentially expressed genes in the adrenal glands of the ISIAH and WAG rats. (PDF 88 kb)
Additional file 3: The genes with the detected expression in adrenal gland of only one rat strain. (XLS 17 kb)
Additional file 4: Functional annotation of differentially expressed genes (DEGs) found in 3-month old ISIAH and WAG adrenal glands. (XLS 148 kb)
Additional file 5: Gene Ontology (GO) terms specifying the main GO terms. (JPG 500 kb)
Additional file 6: Differentially expressed genes (DEGs) in GO term groups, which might be important for the development of the stress-sensitive hypertension in ISIAH rats. (XLS 303 kb)
Additional file 7: KEGG metabolic pathways enriched with genes differentially expressed in 3-month old ISIAH and WAG adrenal glands. (XLS 52 kb)
Additional file 8: The comparison of the relative mRNA abundance between the RNA-Seq and qPCR measurements. (JPG 261 kb)
Additional file 9: The summary statistics for the sequenced libraries. (XLS 14 kb)
Additional file 10: Primers used in qPCR. (DOC 41 kb)

## Abbreviations

BP: Blood pressure
DAVID: Database for annotation, visualization and integrated discovery
DEG: Differentially expressed genes
FDR: False discovery rate
FPKM: Fragments per kilobase of transcript per million mapped reads
GO: Gene ontology
HPA: Hypothalamic-pituitary-adrenal
ISIAH: Inherited stress-induced arterial hypertension
KEGG: Kyoto encyclopedia of genes and genomes pathway database
PLS-DA: Partial-least squares discriminant analysis
qPCR: Quantitative real time polymerase chain reaction
RAS: Renin–angiotensin system
RGD: Rat genome database
RNA-Seq: RNA sequencing
RT: Reverse transcription
SAS: Sympathoadrenal system
sEH: Soluble epoxide hydrolase
WAG: Wistar Albino Glaxo
