# Supplementary figures and images for: Molecular determinants of the adrenal gland functioning related to stress-sensitive hypertension in ISIAH rats
Source: BMC Genomics. 2016 Dec 28;17(Suppl 14):989. doi: 10.1186/s12864-016-3354-2 (PMC5249038; doi:10.1186/s12864-016-3354-2)

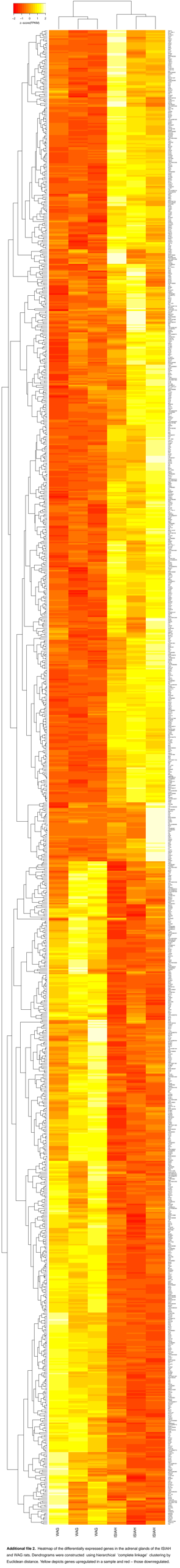

Supplement: Additional file 2: — Heatmap of the differentially expressed genes in the adrenal glands of the ISIAH and WAG rats. (PDF 88 kb) [file 12864_2016_3354_MOESM2_ESM.pdf]

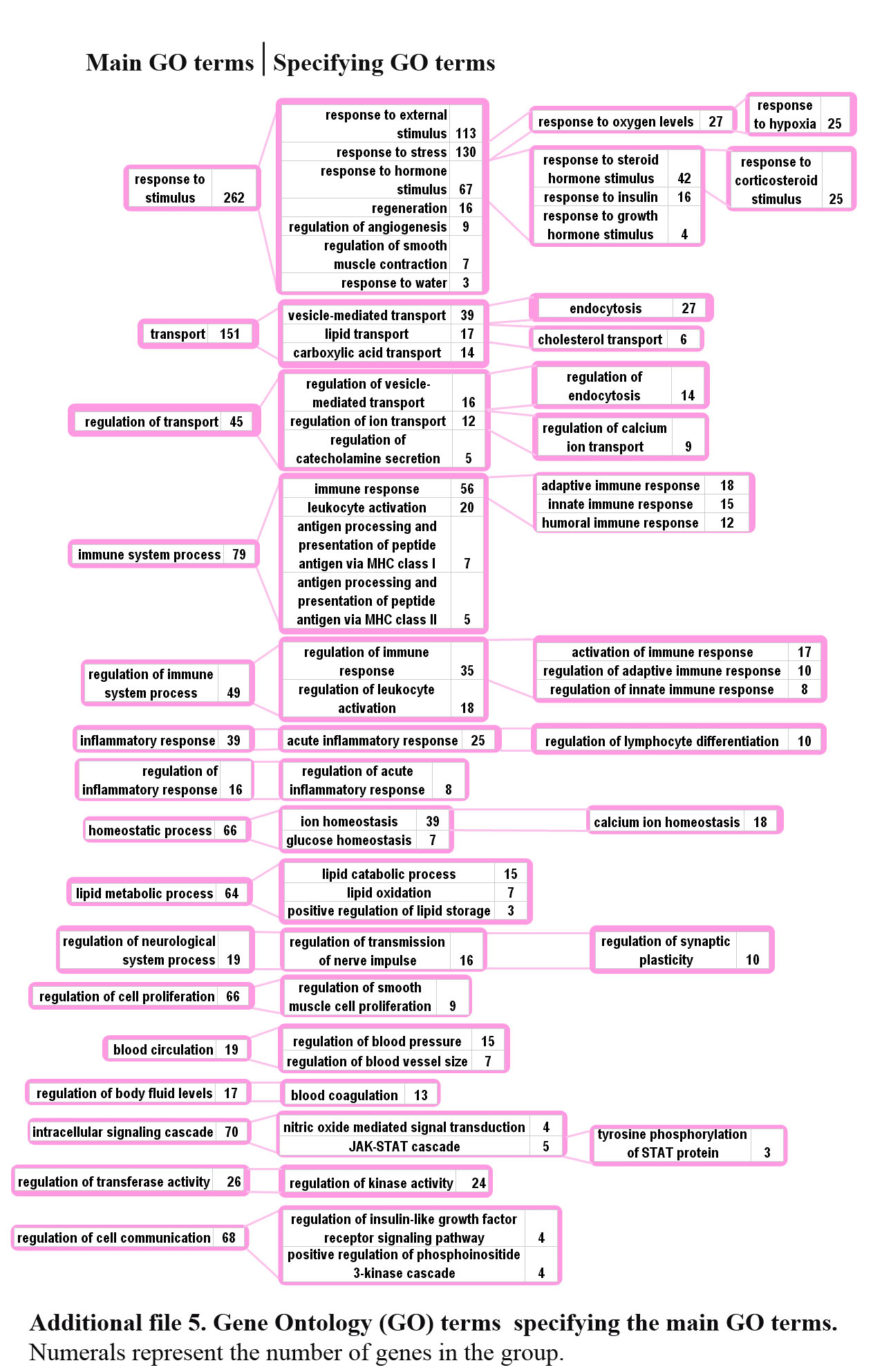

Supplement: Additional file 5: — Gene Ontology (GO) terms specifying the main GO terms. (JPG 500 kb) [file 12864_2016_3354_MOESM5_ESM.jpg]

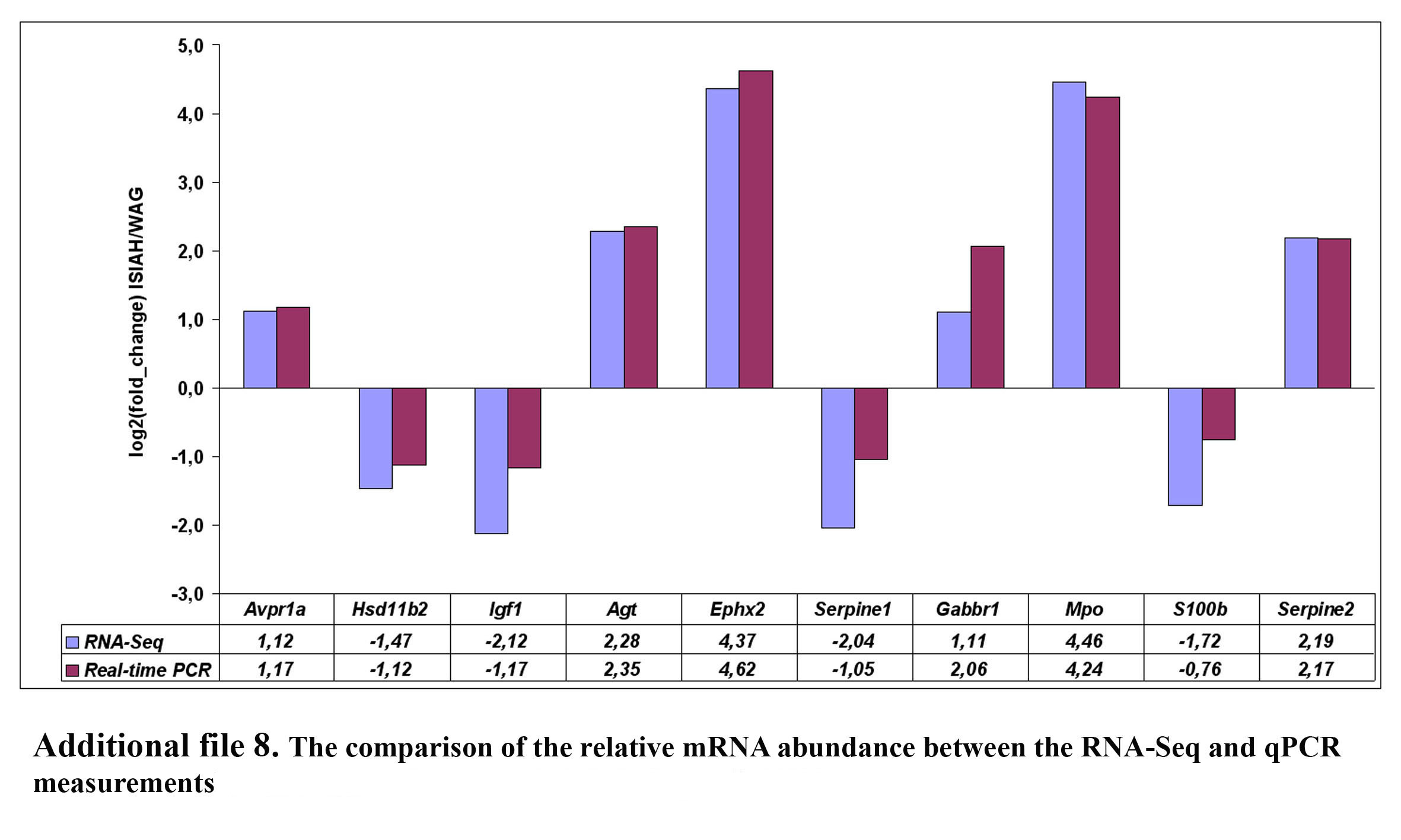

Supplement: Additional file 8: — The comparison of the relative mRNA abundance between the RNA-Seq and qPCR measurements. (JPG 261 kb) [file 12864_2016_3354_MOESM8_ESM.jpg]
