# Supplementary material for: Molecular determinants of the adrenal gland functioning related to stress-sensitive hypertension in ISIAH rats
Source: BMC Genomics. 2016 Dec 28;17(Suppl 14):989. doi: 10.1186/s12864-016-3354-2 (PMC5249038; doi:10.1186/s12864-016-3354-2)
Supplement: Additional file 10: — Primers used in qPCR. (DOC 41 kb) [file 12864_2016_3354_MOESM10_ESM.doc]

**Additional file 10.**

Primers used in real-time PCR

| Gene | Primers, 5’--->3’ | | Annealing temperature, ^o^C | temperature of fluorescence signal acquisition |
| --- | --- | --- | --- | --- |
|  | forward | reverse |  |  |
| *Agt* | CCTCGCTCTCTGGACTTATC | CAGACACTGAGGTGCTGTTG | 64 | 87 |
| *Avpr1a* | CCTTTGTGATTGTAAGCGCC | CAGCATGGGAAACTTTGGAC | 64 | 85 |
| *Ephx2* | TTTCTTGGAGGTACCAGATCC | CAGTCATGGCCAATGAACAC | 62 | 83 |
| *Gabbr1* | CGAACCATTGAGACTTTTGCC | GACACGCTCTTGGTTTCGTA | 62 | 85 |
| *Hsd11b2* | CAAGACAGAGGCAGTGACTAAT | CCCATGCAAGTGCTCAATG | 62 | 84 |
| *Igf1* | GCTTTTACTTCAACAAGCCCA | GGAGCACAGTACATCTCCAG | 63 | 85 |
| *Mpo* | CGCCCAACAACATTGACATC | TCTCCCACCAAAACCTATCG | 63 | 87 |
| *S100b* | CTTCCTGGAGGAAATCAAAGAG | CATGGAGACGAAGGCCATAAA | 63 | 83 |
| *Serpine1* | AAAGGTCAAGATCGAGGTGAA | GAAGAGGATTGTCTCTGTTGGA | 63 | 86 |
| *Serpine2* | AGTGACAACTGCAATCCTAAT | GAGTCTCTCTTCGGAGCAAA | 62 | 86 |
| *Ppia* | TTCCAGGATTCATGTGCCAG | CTTGCCATCCAGCCACTC | 62-64 | 85 |
